# Supplementary material for: Understanding the interaction of 14‐3‐3 proteins with hDMX and hDM2: a structural and biophysical study
Source: FEBS J. 2022 Mar 28;289(17):5341–58. doi: 10.1111/febs.16433 (PMC9541495; doi:10.1111/febs.16433)
Supplement: Supplementary file 1 — Fig. S1. Sequence alignment of 14‐3‐3 proteins. Fig. S2. hDMX and hDM2 sequences. Fig. S3. Sequence alignment for hDM2 and hDMX proteins. Fig. S4. Fluorescence anisotropy assays for the hDM2 peptides. Fig. S5. Fluorescence anisotropy assays for the hDMX144‐158 pThr151 peptide with all isoforms of 14‐3‐3. Fig. S6. Fluorescence anisotropy assays for unphosphorylated hDMX and hDM2 peptides with 14‐3‐3η. Fig. S7. ITC data for the interaction of hDMX and hDM2 peptides with 14‐3‐3η. Fig. S8. Dose–response SPR experiments for the binding of hDMX and hDM2 peptides to immobilized 14‐3‐3η. Fig. S9. Data Fitting for dose–response SPR experiments for the binding of hDMX and hDM2 peptides to immobilized 14‐3‐3η. Fig. S10. AUC data for 14‐3‐3η and hDMX335‐374 pSer342/pSer367. Fig. S11. Additional images for the hDMX361‐374 pSer367/1433σ structure (PDB: 6YR5). Fig. S12. Additional images for the hDM2180‐192 pSer186/1433σ structure (PDB: 6YR6). Fig. S13. hDMX335‐374 pSer342/pSer367/1433σ structure (PDB ID: 6YR7). Table S1. High‐resolution mass spectrometry data for peptides. [file FEBS-289-5341-s001.zip › febs16433-sup-0001-Supinfo.pdf]

FEBS PRESS
science publishing by scientists

WILEY

The FEBS Journal

www.febsjournal.org

Understanding the interaction of 14-3-3 proteins with *h*DMX and *h*DM2: a structural and biophysical study

Sonja Srdanović, Madita Wolter, Chi H. Trinh, Christian Ottmann, Stuart L. Warriner and Andrew J. Wilson

DOI: 10.1111/febs.16433

Understanding the interaction of 14-3-3 proteins with *h*DMX and *h*DM2: a structural and biophysical study

Sonja Srdanovic,[a,b] Madita Wolter,[c,d] Chi H. Trinh,[a,e] Christian Ottmann,[c,d] Stuart L. Warriner,[a,b] Andrew J. Wilson,[a,b]*

[a]Astbury Centre for Structural Molecular Biology, University of Leeds, Woodhouse Lane, Leeds LS2 9JT, UK

[b]School of Chemistry, University of Leeds, Woodhouse Lane, Leeds LS2 9JT, UK

[c]Laboratory of Chemical Biology, Department of Biomedical Engineering, Technische Universiteit Eindhoven, Eindhoven, 5600 MB, The Netherlands

[d]Institute for Complex Molecular Systems, Technische Universiteit Eindhoven, Eindhoven, 5600 MB, The Netherlands

[e]School of Molecular and Cellular Biology, University of Leeds, Woodhouse Lane, Leeds LS2 9JT, UK

*e-mail: a.j.wilson@leeds.ac.uk,

Supporting Information

Contents

Additional Figures 2

Peptide and protein analytical characterization 11

Additional Figures

Protein Sequences and Alignment

```
P27348 1433T_HUMAN          1    --MEKTELIQKAKLAEQAERYDDMATCMKAVTEQGAELSNEERNLLSVAYKNVVGGRRSA       58
P31946 1433B_HUMAN          1    MTMDKSELVQKAKLAEQAERYDDMAAAMKAVTEQGHELSNEERNLLSVAYKNVVGARRSS       60
P61981 1433G_HUMAN          1    -MVDREQLVQKARLAEQAERYDDMAAAMKNVTELNEPLSNEERNLLSVAYKNVVGARRSS       59
P62258 1433E_HUMAN          1    -MDDREDLVYQAKLAEQAERYDEMVESMKKVAGMDVELTVEERNLLSVAYKNVIGARRAS       59
P63104 1433Z_HUMAN          1    --MDKNELVQKAKLAEQAERYDDMAACMKSVTEQGAELSNEERNLLSVAYKNVVGARRSS       58
Q04917 1433F_HUMAN          1    -MGDREQLLQRARLAEQAERYDDMASAMKAVTELNEPLSNEDRNLLSVAYKNVVGARRSS       59
P31947 1433S_HUMAN          1    --MERASLIQKAKLAEQAERYEDMAAFMKGAVEKGEELSCEERNLLSVAYKNVVGGQRAA       58
                                    :: .*: :*:*********::*.  **  ..   .   *: *:***********:*.:*::

P27348 1433T_HUMAN         59    WRVISSIEQKT--DTSDKKLQLIKDYREKVESELRSICTTVLELLDKYLIANAT--NPES      114
P31946 1433B_HUMAN         61    WRVISSIEQKT--ERNEKKQQMGKEYREKIEAELQDICNDVLELLDKYLIPNAT--QPES      116
P61981 1433G_HUMAN         60    WRVISSIEQKTSADGNEKKIEMVRAYREKIEKELEAVCQDVLSLLDNYLIKNCSETQYES      119
P62258 1433E_HUMAN         60    WRIISSIEQKEENKGGEDKLKMIREYRQMVETELKLICCDILDVLDKHLIPAAN--TGES      117
P63104 1433Z_HUMAN         59    WRVVSSIEQKT--EGAEKKQQMAREYREKIETELRDICNDVLSLLEKFLIPNAS--QAES      114
Q04917 1433F_HUMAN         60    WRVISSIEQKTMADGNEKKLEKVKAYREKIEKELETVCNDVLSLLDKFLIKNCNDFQYES      119
P31947 1433S_HUMAN         59    WRVLSSIEQKSNEEGSEEKGPEVREYREKVETELQGVCDTVLGLLDSHLIKEAG--DAES      116
                                 **::******    .  :.*     : **: :* **. :*  :* :*:..**  .      **

P27348 1433T_HUMAN        115    KVFYLKMKGDYFRYLAEVACGDDRKQTIDNSQGAYQEAFDISKKEMQPTHPIRLGLALNF      174
P31946 1433B_HUMAN        117    KVFYLKMKGDYFRYLSEVASGDNKQTTVSNSQQAYQEAFEISKKEMQPTHPIRLGLALNF      176
P61981 1433G_HUMAN        120    KVFYLKMKGDYYRYLAEVATGEKRATVVESSEKAYSEAHEISKEHMQPTHPIRLGLALNY      179
P62258 1433E_HUMAN        118    KVFYYKMKGDYHRYLAEFATGNDRKEAAENSLVAYKAASDIAMTELPPTHPIRLGLALNF      177
P63104 1433Z_HUMAN        115    KVFYLKMKGDYYRYLAEVAAGDDKKGIVDQSQQAYQEAFEISKKEMQPTHPIRLGLALNF      174
Q04917 1433F_HUMAN        120    KVFYLKMKGDYYRYLAEVASGEKKNSVVEASEAAYKEAFEISKEQMQPTHPIRLGLALNF      179
P31947 1433S_HUMAN        117    RVFYLKMKGDYYRYLAEVATGDDKKRIIDSARSAYQEAMDISKKEMPPTNPIRLGLALNF      176
                                 :*** ******.***:*.* *:.:     . :  **. * :*:  .: **:*********:

P27348 1433T_HUMAN        175    SVFYYEILNNPELACTLAKTAFDEAIAELDTLNEDSYKDSTLIMQLLRDNLTLWTSDSAG      234
P31946 1433B_HUMAN        177    SVFYYEILNSPEKACSLAKTAFDEAIAELDTLNEESYKDSTLIMQLLRDNLTLWTSENQG      236
P61981 1433G_HUMAN        180    SVFYYEIQNAPEQACHLAKTAFDDAIAELDTLNEDSYKDSTLIMQLLRDNLTLWTSDQQD      239
P62258 1433E_HUMAN        178    SVFYYEILNSPDRACRLAKAAFDDAIAELDTLSEESYKDSTLIMQLLRDNLTLWTSDMQG      237
P63104 1433Z_HUMAN        175    SVFYYEILNSPEKACSLAKTAFDEAIAELDTLSEESYKDSTLIMQLLRDNLTLWTSDTQG      234
Q04917 1433F_HUMAN        180    SVFYYEIQNAPEQACLLAKQAFDDAIAELDTLNEDSYKDSTLIMQLLRDNLTLWTSDQQD      239
P31947 1433S_HUMAN        177    SVFHYEIANSPEEAISLAKTTFDEAMADLHTLSEDSYKDSTLIMQLLRDNLTLWTADNAG      236
                                 ***:*** * *: *  *** :**:*:*:*.**.*:*******************::  .

P27348 1433T_HUMAN        235    EECDAAEGAEN-------                                                 245
P31946 1433B_HUMAN        237    DEGDAGEGEN--------                                                 246
P61981 1433G_HUMAN        240    DDGGEGNN----------                                                 247
P62258 1433E_HUMAN        238    DGEEQNKEALQDVEDENQ                                                 255
P63104 1433Z_HUMAN        235    DEAEAGEGGEN-------                                                 245
Q04917 1433F_HUMAN        240    EEAGEGN-----------                                                 246
P31947 1433S_HUMAN        237    EEGGEAPQEPQS------                                                 248
                                 :
```

Figure S1. Sequence alignment of 14-3-3 Proteins**.** Sequences were derived from the UniProt server with the following accession codes for the seven human 14-3-3 isoforms: τ- P27348, η-Q04917, σ- P31947, ε-P62258, β- P31946, γ- P61981, ζ- P63104, and aligned using Clustal Omega[1]; conserved amino acids are in dark grey whilst similar amino acids are in light grey**.**

UniProt code: O15151,*h*DMX_HUMAN Protein

```
MTSFSTSAQCSTSDSACRISPGQINQVRPKLPLLKILHAAGAQGEMFTVKEVMHYLGQYI
MVKQLYDQQEQHMVYCGGDLLGELLGRQSFSVKDPSPLYDMLRKNLVTLATATTDAAQTL
ALAQDHSMDIPSQDQLKQSAEESSTSRKRTTEDDIPTLPTSEHKCIHSREDEDLIENLAQ
DETSRLDLGFEEWDVAGLPWWFLGNLRSNYTPRSNGSTDLQTNQDVGTAIVSDTTDDLWF
LNESVSEQLGVGIKVEAADTEQTSEEVGKVSDKKVIEVGKNDDLEDSKSLSDDTDVEVTS
EDEWQCTECKKFNSPSKRYCFRCWALRKDWYSDCSKLTHSLSTSDITAIPEKENEGNDVP
DCRRTISAPVVRPKDAYIKKENSKLFDPCNSVEFLDLAHSSESQETISSMGEQLDNLSEQ
RTDTENMEDCQNLLKPCSLCEKRPRDGNIIHGRTGHLVTCFHCARRLKKAGASCPICKKE
IQLVIKVFIA
```

UniProt code: Q00987, *h*DM2_HUMAN E3 ubiquitin-protein ligase

```
MCNTNMSVPTDGAVTTSQIPASEQETLVRPKPLLLKLLKSVGAQKDTYTMKEVLFYLGQY
IMTKRLYDEKQQHIVYCSNDLLGDLFGVPSFSVKEHRKIYTMIYRNLVVVNQQESSDSGT
SVSENRCHLEGGSDQKDLVQELQEEKPSSSHLVSRPSTSSRRRAISETEENSDELSGERQ
RKRHKSDSISLSFDESLALCVIREICCERSSSSESTGTPSNPDLDAGVSEHSGDWLDQDS
VSDQFSVEFEVESLDSEDYSLSEEGQELSDEDDEVYQVTVYQAGESDTDSFEEDPEISLA
DYWKCTSCNEMNPPLPSHCNRCWALRENWLPEDKGKDKGEISEKAKLENSTQAEEGFDVP
DCKKTIVNDSRESCVEENDDKITQASQSQESEDYSQPSTSSSIIYSSQEDVKEFEREETQ
DKEESVESSLPLNAIEPCVICQGRPKNGCIVHGKTGHLMACFTCAKKLKKRNKPCPVCRQ
PIQMIVLTYFP
```

Figure S2 *h*DMX and *h*DM2 sequences; highlighting serine and threonine residues (bold and blue).

```
Q00987 MDM2_HUMAN          1    MCNTNMSVPTDGAVTTSQIPASEQETLVRPKPLLLKLLKSVGAQKDTYTMKEVLFYLGQY
O15151 MDM4_HUMAN          1    MTSFSTSAQCSTSDS-ACRISPGQINQVRPKLPLLKILHAAGAQGEMFTVKEVMHYLGQY
                                *  .  . *.   . : : :    :  *  . ****  ***:*::.*** : :*:***:.*****

Q00987 MDM2_HUMAN         61    IMTKRLYDEKQQHIVYCSNDLLGDLFGVPSFSVKEHRKIYTMIYRNLVVVNQQESSDSGT
O15151 MDM4_HUMAN         60    IMVKQLYDQQEQHMVYCGGDLLGELLGRQSFSVKDPSPLYDMLRKNLVTLATATTDAAQT
                             **.*:***:::**:***..****:*:*  *****:    :* *: :***.:      :. : *

Q00987 MDM2_HUMAN        121    SVSENRCHLEGGSDQKDLVQELQEEKPS-----SSHLVSRPSTSSRRRAISETEENSDEL
O15151 MDM4_HUMAN        120    LALA-QDHSMDIPSQDQLKQSAEESSTSRKRTTEDDIPTLPTS--EHKCIHS-REDEDLI
                              .    : *   .   .*.:* *. :*.. *       ...: : *::  .::.* . .*:.* :

Q00987 MDM2_HUMAN        176    SGERQRKRHKSDSISLSFDES----LALCVI---REICCERSSSSESTGTPSNPDLDAG-
O15151 MDM4_HUMAN        176    ENLA---QDETSRLDLGFEEWDVAGLPWWFLGNLRSNYTPRSNG--STDLQTNQDVGTAI
                             ..      :.::. :.*.*:*       *    .:     *.      **..   **.   :* *:.:.

Q00987 MDM2_HUMAN        228    -VSEHSGDWLDQDSVSDQFSVEFEVESLDSEDYSLSEEGQELSDEDDEVYQVTVYQAGE-
O15151 MDM4_HUMAN        231    VSDTTDDLWFLNESVSEQLGVGIKVEAADTEQTSEE--V-------GKVSDKKVIEVGKN
                               .   .. *: ::***:*:.* ::**: *:*: * .          .:* : .* :.*:

Q00987 MDM2_HUMAN        286    -----SDTDSFEEDPEISLADYWKCTSCNEMNPPLPSHCNRCWALRENWLPEDKGKDKGE
O15151 MDM4_HUMAN        282    DDLEDSKSLSDDTDVEVTSEDEWQCTECKKFNSPSKRYCFRCWALRKDWYSDCSKLTHS-
                                  *.: * : * *::  * *:**.*:::* *   :* ******::*  : .   :.

Q00987 MDM2_HUMAN        341    ISEKAKLENSTQAEEGFDVPDCKKTIVNDSR---ESCVEENDDKITQASQSQESEDYSQP
O15151 MDM4_HUMAN        341    LSTSDITAIPEKENEGNDVPDCRRTISAPVVRPKDAYIKKENSKLFDPCNSVEFLDLAHS
                             :* .         : :** *****::**        :: :::::.*: : .:* *  * ::

Q00987 MDM2_HUMAN        398    STSSSIIYSSQEDVKEFEREETQDKEESVESSLPLNAIEPCVICQGRPKNGCIVHGKTGH
O15151 MDM4_HUMAN        401    SESQETISSMGEQLDNLSEQR----TDTENMEDCQNLLKPCSLCEKRPRDGNIIHGRTGH
                             * *.. * *  *::.::..:.     :: : .   * ::** :*: **::* *:**:***

Q00987 MDM2_HUMAN        458    LMACFTCAKKLKKRNKPCPVCRQPIQMIVLTYFP
O15151 MDM4_HUMAN        457    LVTCFHCARRLKKAGASCPICKKEIQLVIKVFIA
                             *::** **::*** .  **:*:: **::: .::
```

Figure S3. Sequence alignment for *h*DM2 and *h*DMX proteins. Sequences of the human proteins *h*DM2 (accession code Q00987) and *h*DMX (accession code O15151) were derived from the UniProt server and aligned using Clustal Omega[1]; conserved amino acids are in dark grey whilst similar amino acids are in light grey.

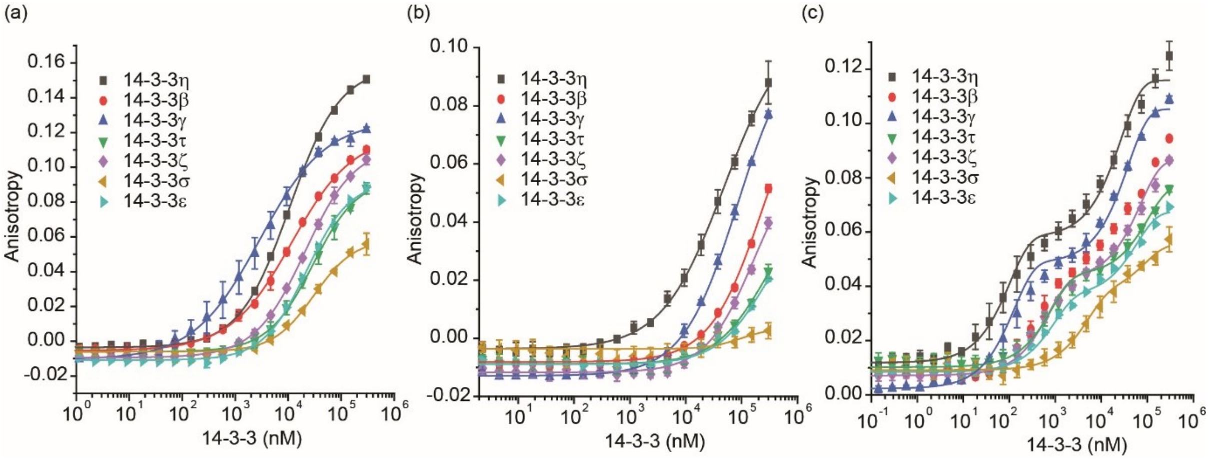

Figure S4. Fluorescence anisotropy assays for the *h*DM2 peptides. (a) *h*DM2$_{160\text{-}171}$^{pSer166} and *h*DM2$_{180\text{-}192}$^{pSer186} (c) *h*DM2$_{160\text{-}192}$$^{pSer166/pSer186}$ with all isoforms of 14-3-3 (FAM tracer peptide 50 nM, 0.1 nM - 300 μM protein, in 10 mM HEPES, 150 mM NaCl, 0.1% Tween 20, 0.1% BSA pH 7.4, concentration of proteins given as 14-3-3 monomer concentration, error bars represent SD for n = 3 replicates).

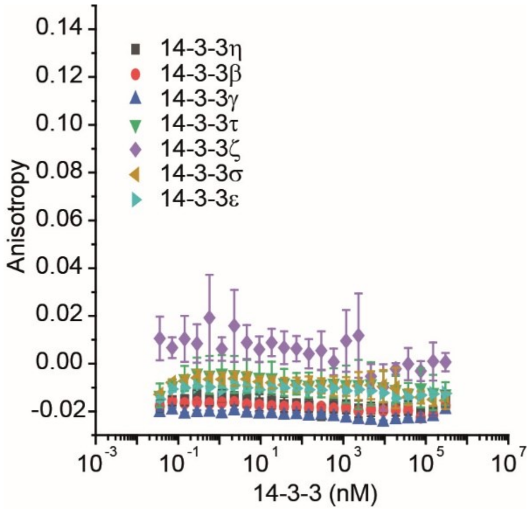

Figure S5. Fluorescence anisotropy assays for the *h*DMX$_{144\text{-}158}$^{pThr151} peptide with all isoforms of 14-3-3. FAM tracer peptide 50 nM, 0.1 nM - 300 μM protein, in 10 mM HEPES, 150 mM NaCl, 0.1% Tween 20, 0.1% BSA pH 7.4, concentration of proteins given as 14-3-3 monomer concentration, error bars represent SD for n = 3 replicates.

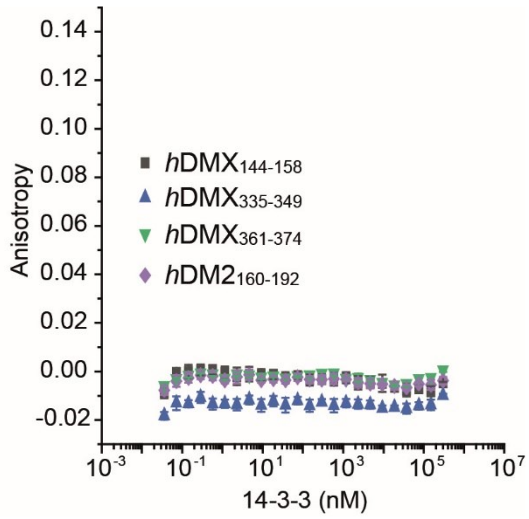

Figure S6. Fluorescence anisotropy assays for unphosphorylated *h*DMX and *h*DM2 peptides with 14-3-3η. FAM tracer peptide 50 nM, 0.1 nM - 300 µM protein, in 10 mM HEPES, 150 mM NaCl, 0.1% Tween 20, 0.1% BSA pH 7.4, concentration of proteins given as 14-3-3 monomer concentration, error bars represent SD for n = 3 replicates.

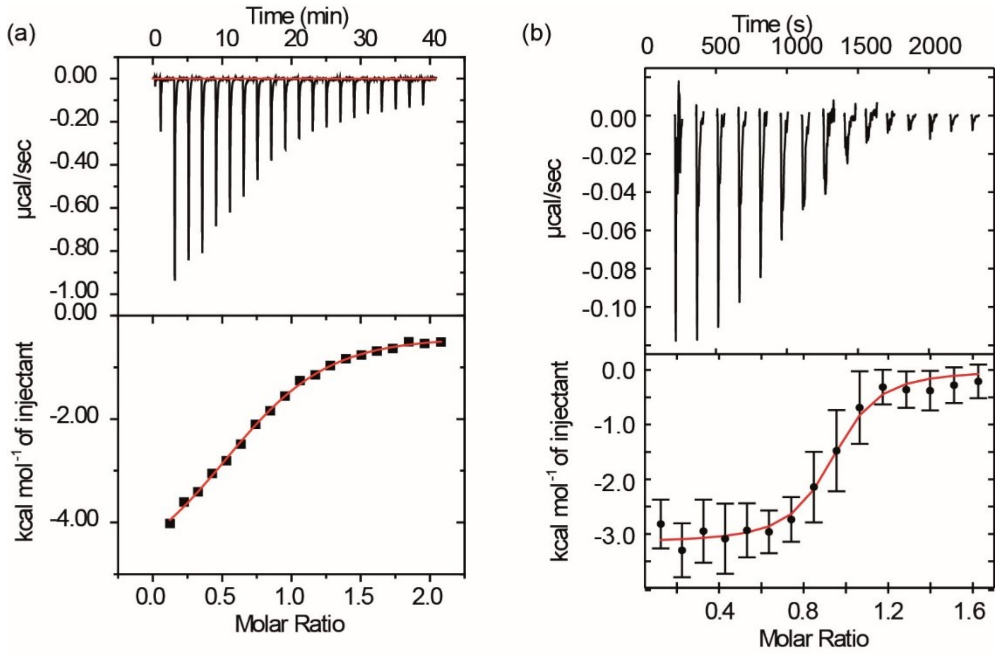

Figure S7. ITC data for the interaction of *h*DMX and *h*DM2 peptides with 14-3-3η; (a) *h*DMX$_{335\text{-}349}^{pSer342}$; (b) *h*DM2$_{160\text{-}192}^{pSer166/pSer186}$ fitted with SEDPHAT (error bars represent estimated integration errors generated by the software NITPIC[2] following singular value decomposition). Peptides were titrated into 14-3-3 η {0.1M for mono phosphorylated peptide and 0.02M for doubly phosphorylated peptide}, 25°C, 25 mM HEPES pH 7.5, 100 mM NaCl, 10 mM $MgCl_2$, 0.5 mM TCEP.

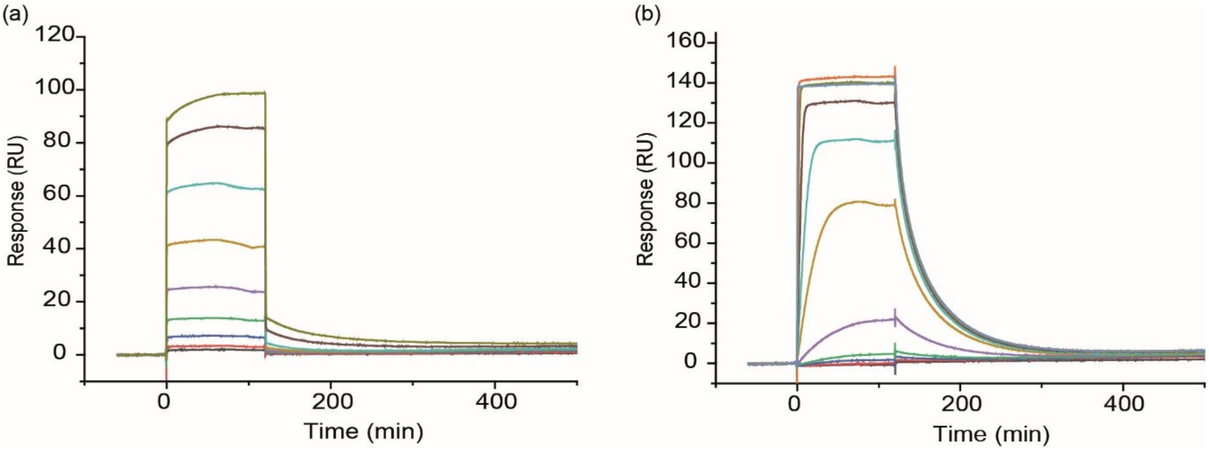

Figure S8. Dose response SPR experiments for the binding of *h*DMX and *h*DM2 peptides to immobilized 14-3-3η; (a) *h*DMX$_{335\text{-}349}$^{pSer342}; (b) *h*DM2$_{160\text{-}192}$$^{pSer166/pSer186}$ (peptides – concentration at 10x the K_d for each peptide – were passed over immobilized 14-3-3η, 25°C, 25 mM HEPES pH 7.5, 100 mM NaCl, 10 mM $MgCl_2$; experiments were performed in a multicycle kinetic format and data was fitted to a Langmuir model. K_d values were determined by fitting maximal response level at the end of injection against protein concentration using a steady state affinity model in the Biocore evaluation software.

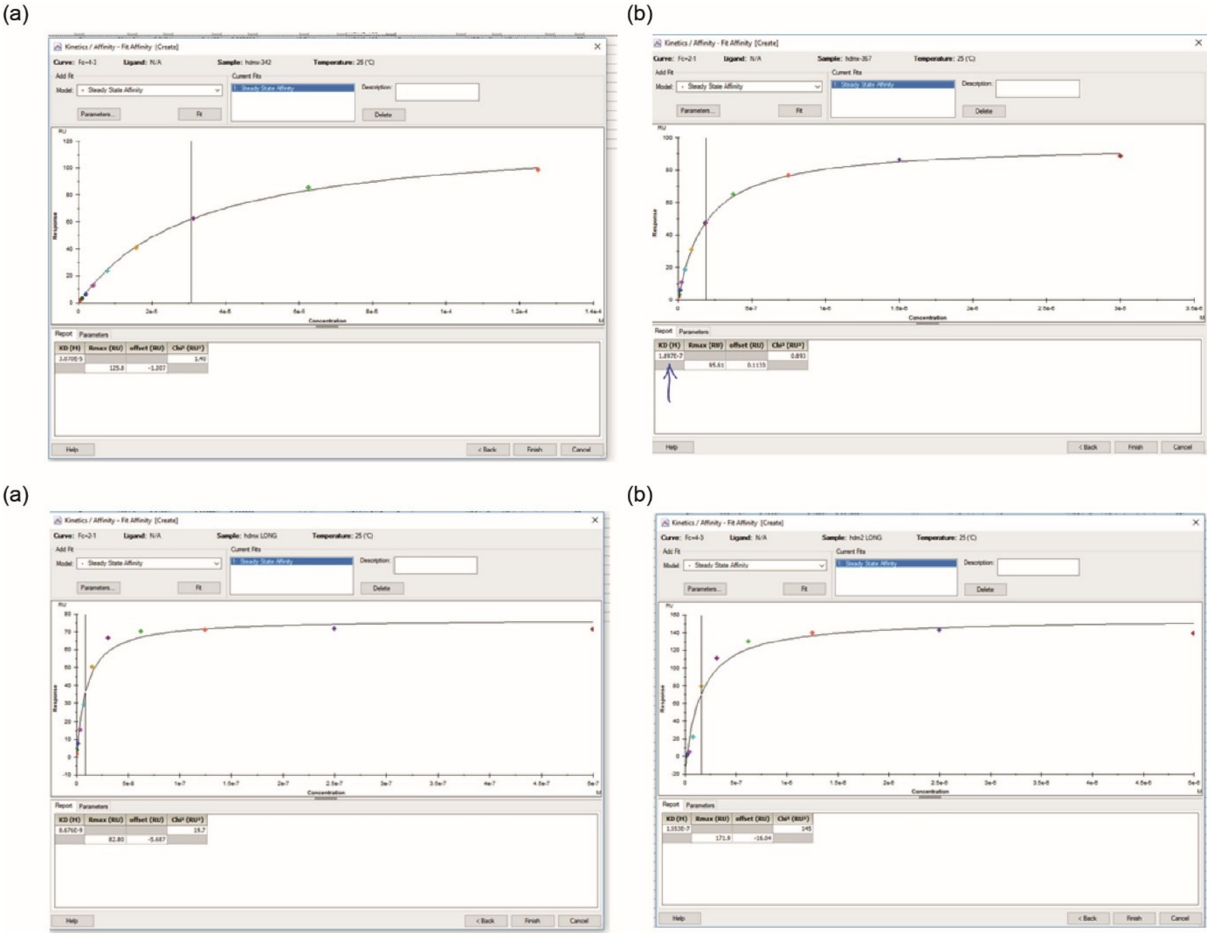

Figure S9. Data Fitting for Dose response SPR experiments for the binding of *h*DMX and *h*DM2 peptides to immobilized 14-3-3η; (a) *h*DMX$_{335\text{-}349}$^{pSer342}; (b) *h*DMX$_{361\text{-}374}$^{pSer367}, (c) *h*DMX$_{335\text{-}373}$$^{pSer342/pSer367}$ and (d) *h*DM2$_{160\text{-}192}$$^{pSer166/pSer186}$.

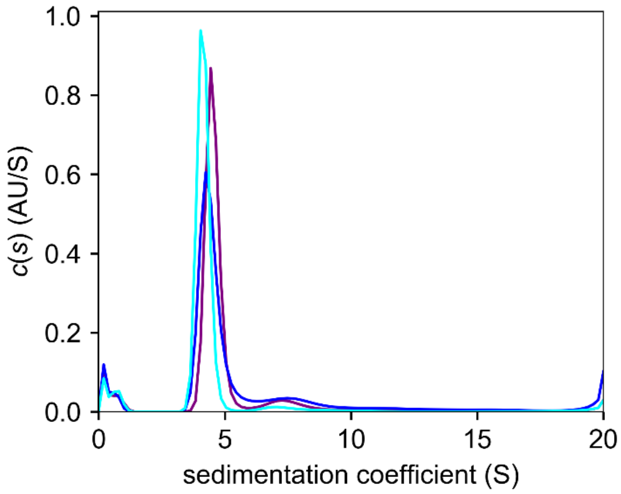

Figure S10. AUC data for 14-3-3η and *h*$DMX_{335-374}^{pSer342/pSer367}$; for 14-3-3η alone (purple) and in a mixture with *h*$DMX_{335-374}^{pSer342/pSer367}$ (0.5 eq dark blue, 1.0 eq. light blue), indicating only one physiological dimer is involved in the binding (14 μM of protein used with two different rations of *h*$DMX_{335-374}^{pSer342/pSer367}$, in 10 mM HEPES, 150 mM NaCl, 0.1% Tween 20).

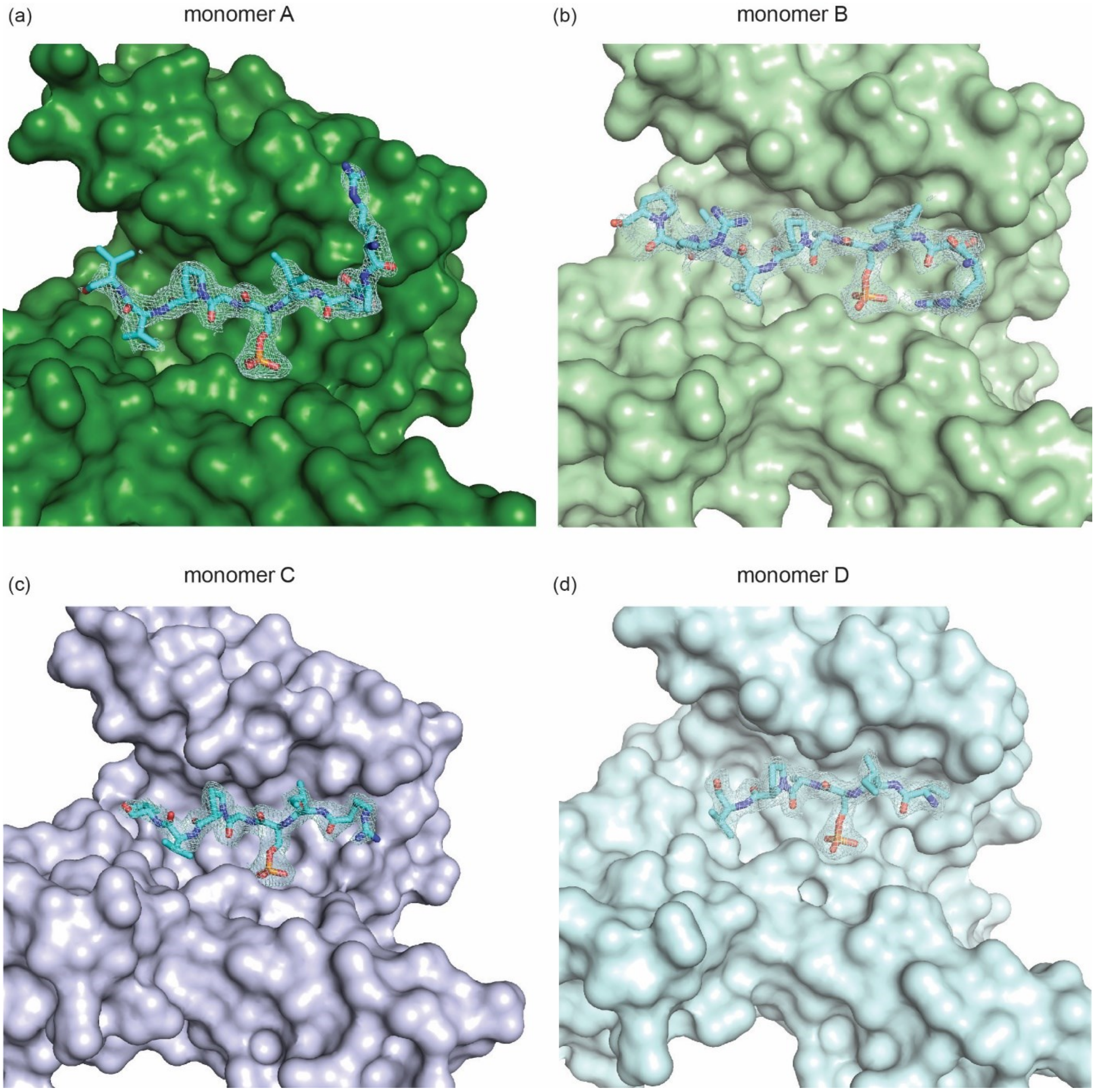

Figure S11 Additional images for the *h*$DMX_{361\text{-}374}^{pSer367}$/14-3-3σ structure (PDB: 6YR5); each panel shows each singly phosphorylated peptide ($2F_o$- F_c electron density map, contoured at 1σ) bound to a 14-3-3σ monomer (dark green, light green, light blue or light lilac surface), in its conserved amphipathic groove (*h*$DMX_{361\text{-}374}^{pSer367}$ shown as sticks, carbon in cyan, phosphorous orange, nitrogen dark blue and oxygen red). Structure images were generated using PyMOL 1.8[3] and collated on an Adobe Illustrator CS6 artboard.

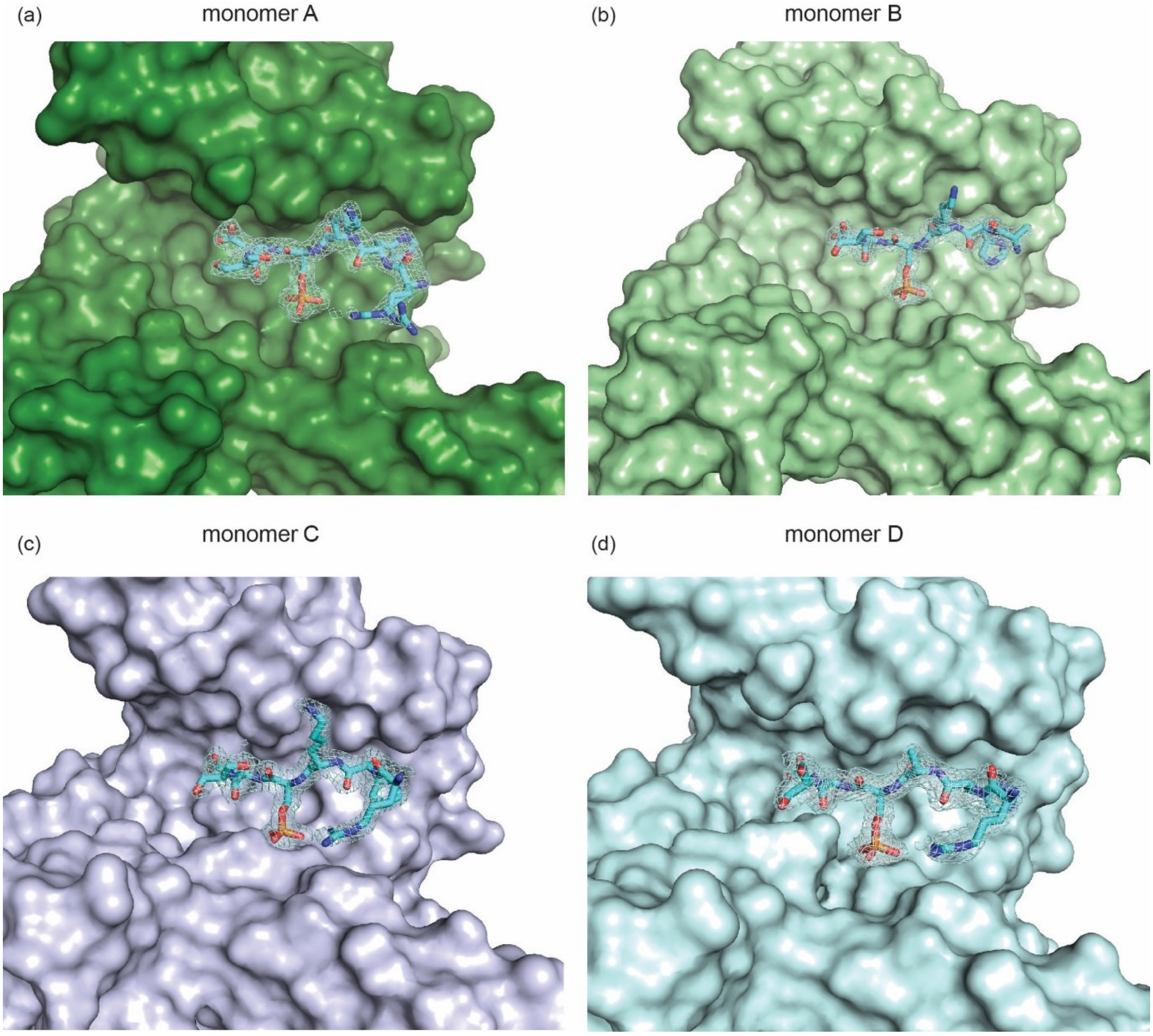

Figure S12 Additional images for the *h*DM2$_{180\text{-}192}^{pSer186}$/14-3-3σ structure (PDB: 6YR6); each panel shows each singly phosphorylated peptide (2F$_o$- F$_c$ electron density map, contoured at 1σ) bound to a 14-3-3σ monomer (dark green, light green, light blue or light lilac surface), in its conserved amphipathic groove (*h*DM2$_{180\text{-}192}^{pSer186}$ shown as sticks, carbon in cyan, phosphorous orange, nitrogen dark blue and oxygen red). Structure images were generated using PyMOL 1.8[3] and collated on an Adobe Illustrator CS6 artboard.

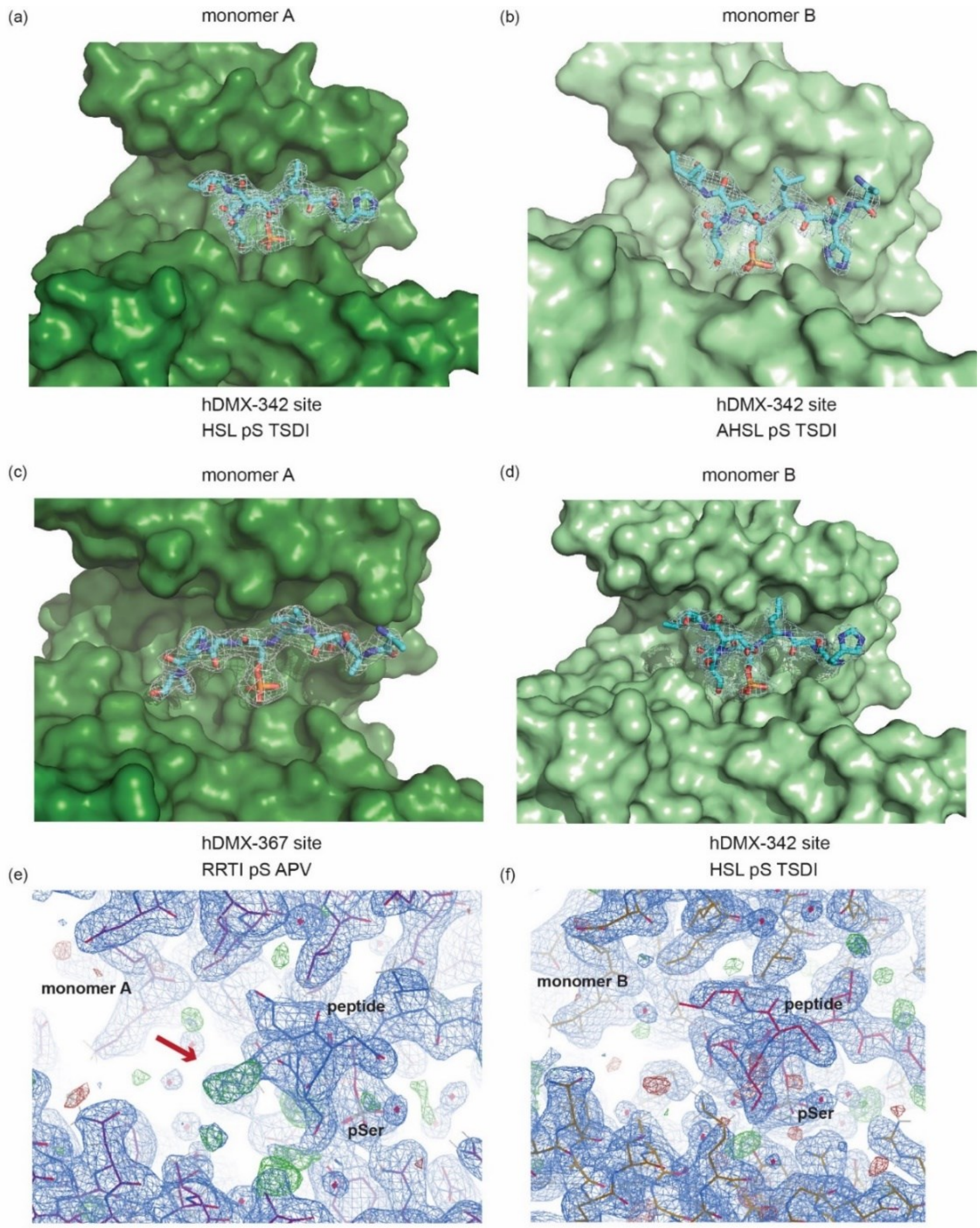

Figure S13 hDMX$_{335\text{-}374}^{pSer342/pSer367}$/14-3-3σ structure (PDB ID: 6YR7); (a-b) show the final refined structure with both pSer342 in 14 3 3σ monomer A and B (dark green, light green surface), in its conserved amphipathic groove (hDMX-342 and hDMX-367 sites shown as sticks with carbon in cyan, phosphorous orange, nitrogen dark blue and oxygen red), (c-d) shows electron density during the refinement, where pSer367 could be in monomer A. (e-f) show electron density for the final refined structure ($2F_o$- F_c electron density map in blue, contoured at 1σ). Extra electron density can be observed in monomer A (F_o- F_c , contoured at 2.5σ, in green) indicating pSer342 and pSer367 peptides overlapping. For monomer A, the dominant electron density map can be assigned to the pSer342 binding site as the unusually bent conformation is not supported by the Pro369 of the second binding site (pSer367). Nevertheless, after modeling in the pSer342 site extra electron density could be observed adjacent to Ser344 of the first binding site of the peptide. This extra electron density fits to the sequence of the pSer342 binding site, confirming the compatibility of the pSer342 with monomer A. Structure images were generated using PyMOL 1.8[3] and collated on an Adobe Illustrator CS6 artboard.

Peptide and protein analytical characterization

Table S1. High resolution mass spectrometry data for peptides

Peptide	$[M+2H]^{1+}$ Obs[d]	$[M+2H]^{1+}$ Exp[d]	$[M+3H]^{2+}$ Obs[d]	$[M+3H]^{2+}$ Exp[d]	$[M+4H]^{3+}$ Obs[d]	$[M+4H]^{3+}$ Exp[d]
*h*DMX$_{144\text{-}158}^{\text{pThr151}}$	920.9451	920.9437	614.2984	614.2982	N/A	460.9755
FAM-Ahx *h*DMX$_{144\text{-}158}^{\text{pThr151}}$	1136.0173	1136.0086	757.6755	757.6748	568.5060	568.5079
FAM-Ahx *h*DMX$_{144\text{-}158}$	1096.0239	1096.0255	731.0186	731.0194	568.5060	548.5163
*h*DMX$_{335\text{-}349}^{\text{pSer342}}$	847.9230	847.9217	565.6169	565.6169	N/A	424.4645
FAM-Ahx *h*DMX$_{335\text{-}349}^{\text{pSer342}}$	1062.9899	1062.9866	708.9934	708.9935	N/A	531.9970
FAM-Ahx *h*DMX$_{335\text{-}349}$	1023.0011	1023.0035	682.3364	682.3381	N/A	512.0054
*h*DMX$_{361\text{-}374}^{\text{pSer367}}$	859.9489	859.9478	573.6359	573.6343	430.4781	430.4776
FAM-Ahx *h*DMX$_{361\text{-}374}^{\text{pSer367}}$	1075.0167	1075.0128	717.0097	717.0109	537.9939	538.0100
FAM-Ahx *h*DMX$_{361\text{-}374}$	1035.0430	1035.0296	690.3517	690.3555	517.9810	518.0185
*h*DM2$_{160\text{-}171}^{\text{pSer166}}$	784.8648	784.8625	523.5776	523.5774	N/A	392.9349
FAM-Ahx *h*DM2$_{160\text{-}171}^{\text{pSer166}}$	999.9097	999.9274	666.9422	666.9540	500.2079	500.4674
*h*DM2$_{180\text{-}192}^{\text{pSer186}}$	831.9259	831.9254	554.9526	554.9527	416.4659	416.4663
FAM-Ahx *h*DM2$_{180\text{-}192}^{\text{pSer186}}$	1047.0032	1046.9904	698.3269	698.3293	523.9629	523.9988
	$[M+3H]^{1+}$ Obs[d]	$[M+3H]^{1+}$ Exp[d]	$[M+4H]^{2+}$ Obs[d]	$[M+4H]^{2+}$ Exp[d]	$[M+5H]^{3+}$ Obs[d]	$[M+5H]^{3+}$ Exp[d]
*h*DMX$_{335\text{-}373}^{\text{pSer342/pSer367}}$	1479.7087	1479.046	1110.5397	1109.5366	888.4359	887.8308
FAM-Ahx *h*DMX$_{335\text{-}373}^{\text{pSer342/pSer367}}$	1622.7495	1622.087	1217.3286	1216.8169	974.0712	973.655
FAM-Ahx *h*DMX$_{345\text{-}373}^{\text{pSer342}}$	1595.7648	1595.431	1197.3266	1196.8254	957.8637	957.6617
FAM-Ahx *h*DMX$_{345\text{-}373}^{\text{pSer367}}$	1595.7632	1595.431	1197.0930	1196.8254	957.8848	957.6617
*h*DM2$_{160\text{-}192}^{\text{pSer166/pSer186}}$	1349.6392	1349.308	1012.7332	1012.2325	810.3888	809.9875
FAM-Ahx *h*DM2$_{160\text{-}192}^{\text{pSer166/pSer186}}$	1493.0156	1492.348	1120.0278	1119.5129	896.2256	895.8117
FAM-Ahx *h*DM2$_{160\text{-}192}$	1439.7003	1439.037	1079.7792	1079.5297	864.0253	863.8252

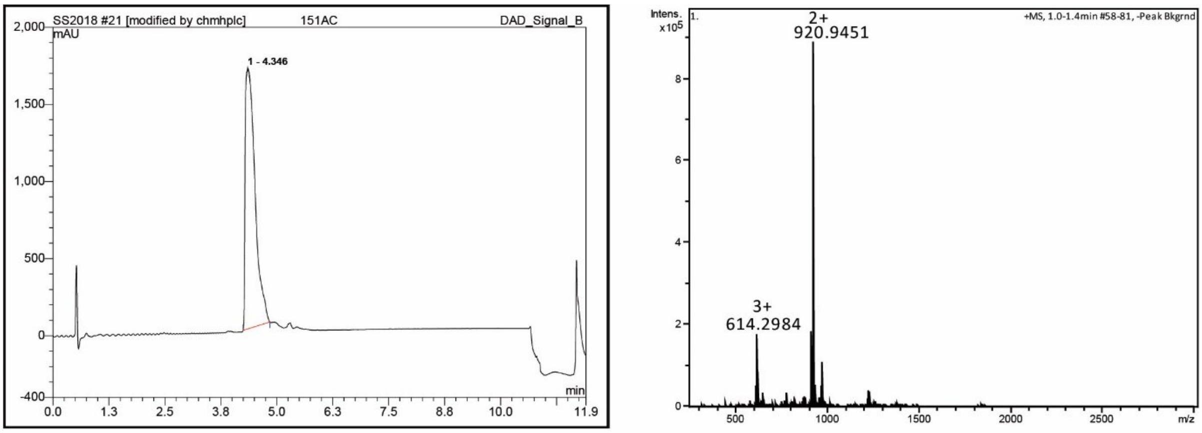

hDMX$_{144\text{-}158}^{pThr151}$

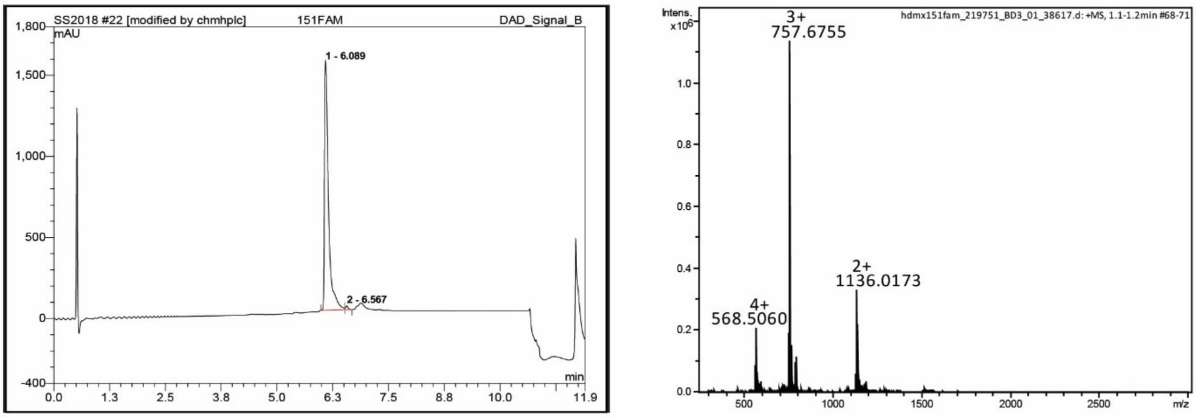

FAM-Ahx hDMX$_{144\text{-}158}^{pThr151}$

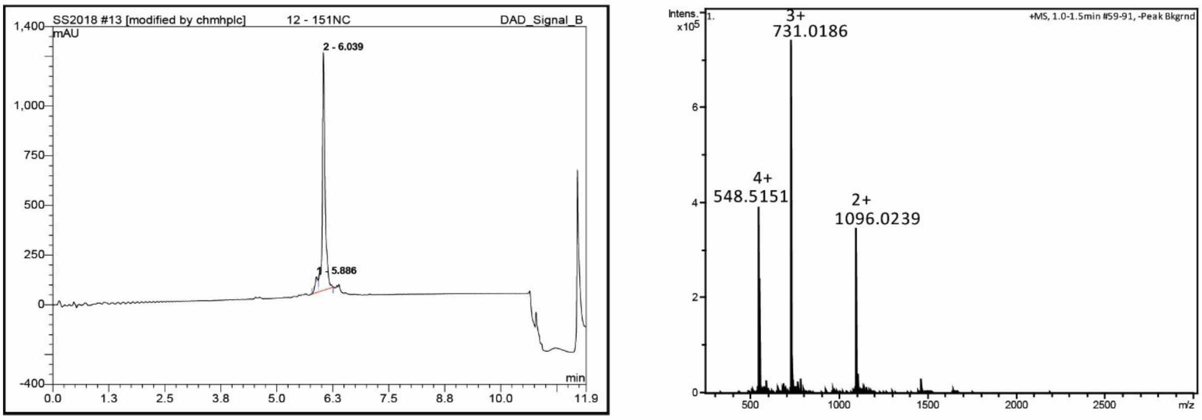

FAM-Ahx hDMX$_{144\text{-}158}$

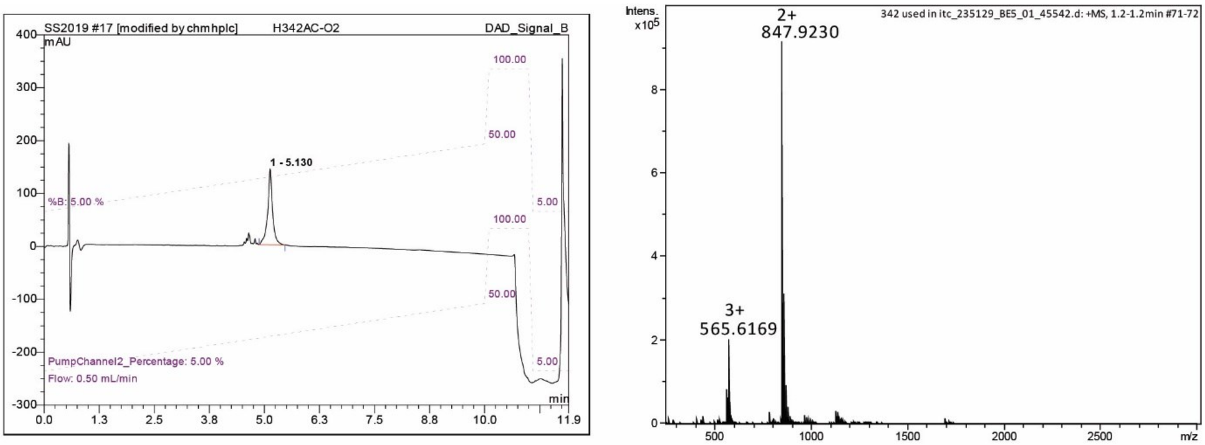

*h*DMX$_{335-349}$^{pSer342}

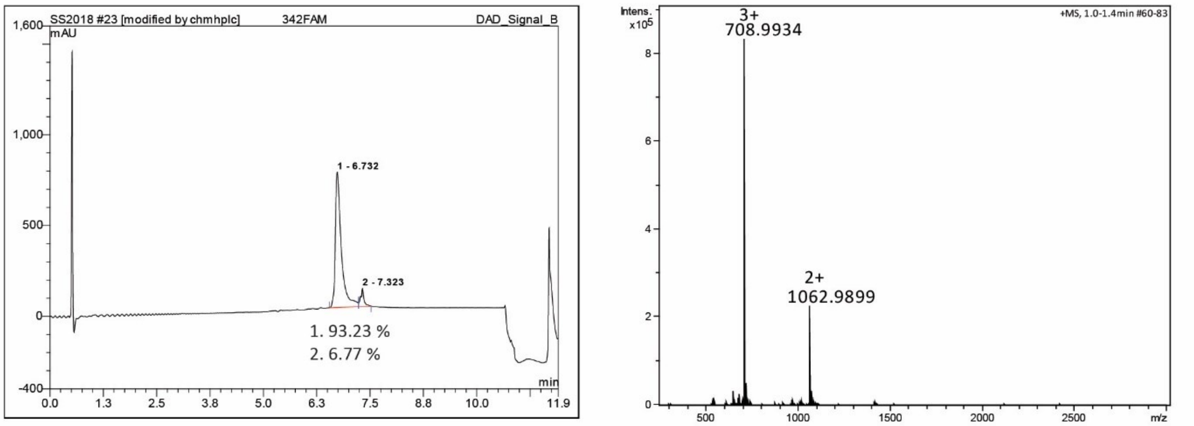

FAM-Ahx *h*DMX$_{335-349}$^{pSer342}

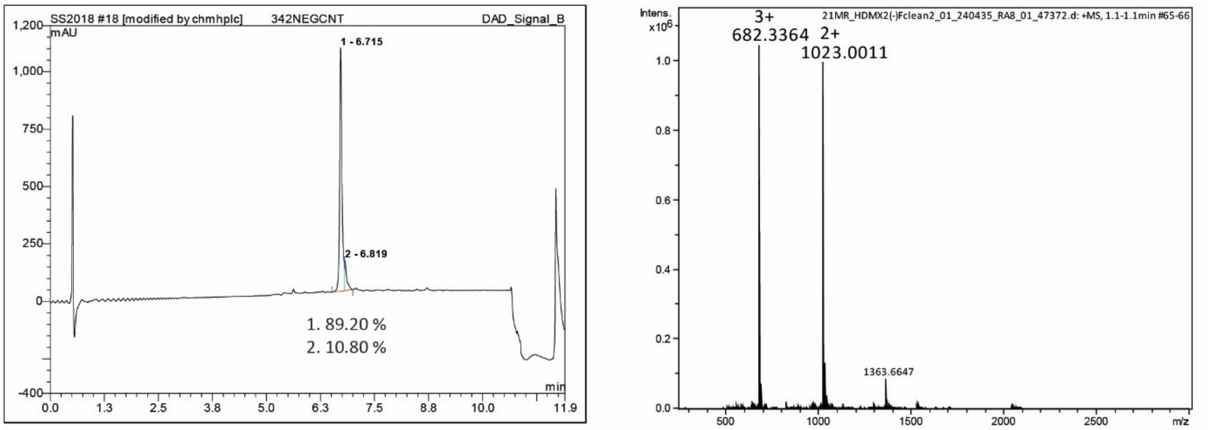

FAM-Ahx *h*DMX$_{335-349}$

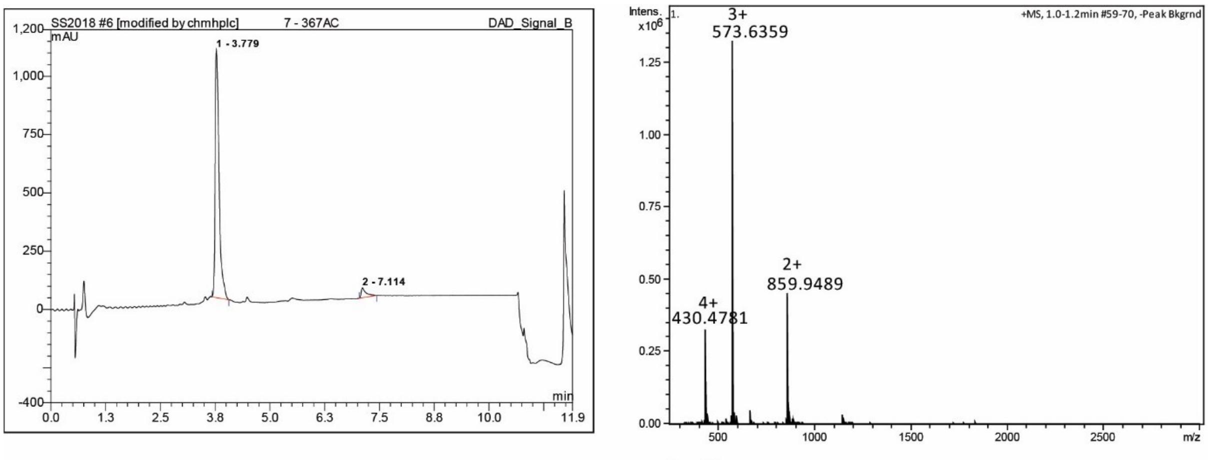

*h*DMX$_{361\text{-}374}^{\text{pSer367}}$

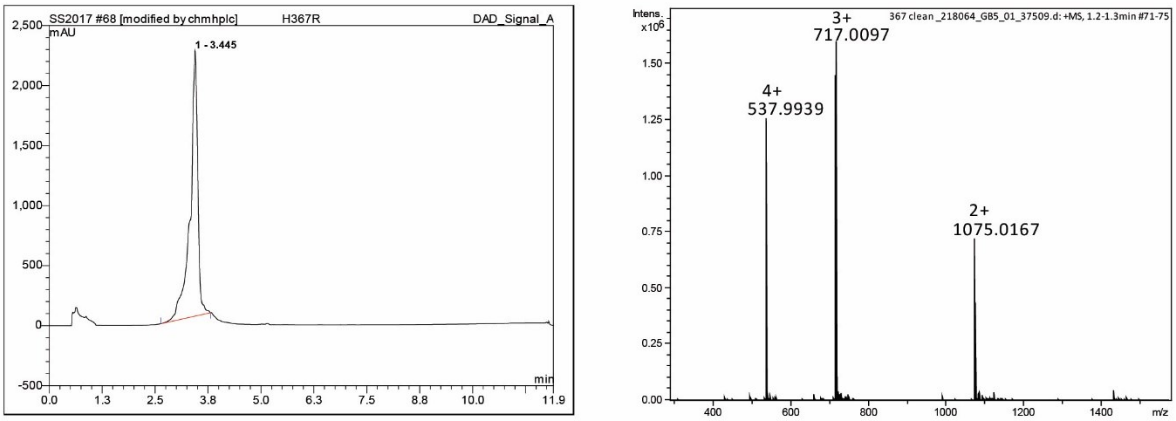

FAM-Ahx *h*DMX$_{361\text{-}374}^{\text{pSer367}}$

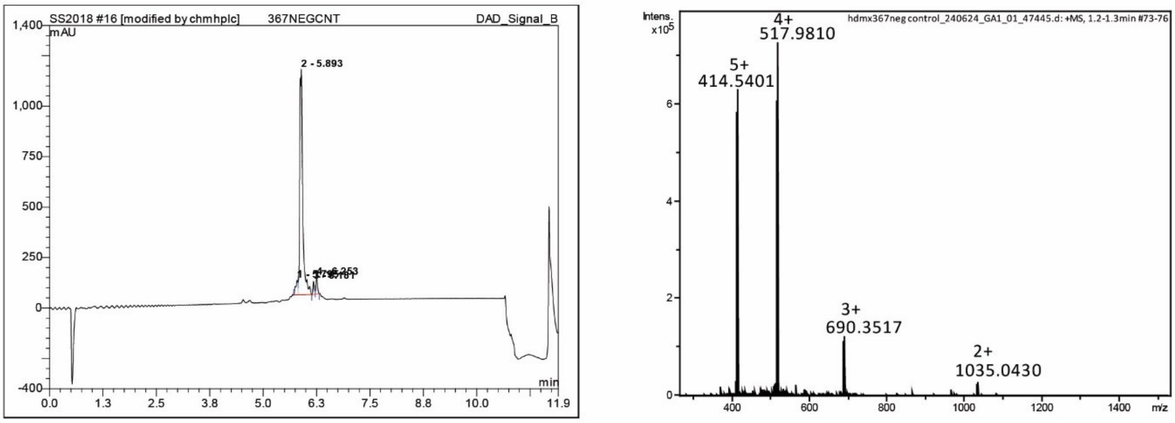

FAM-Ahx *h*DMX$_{361\text{-}374}$

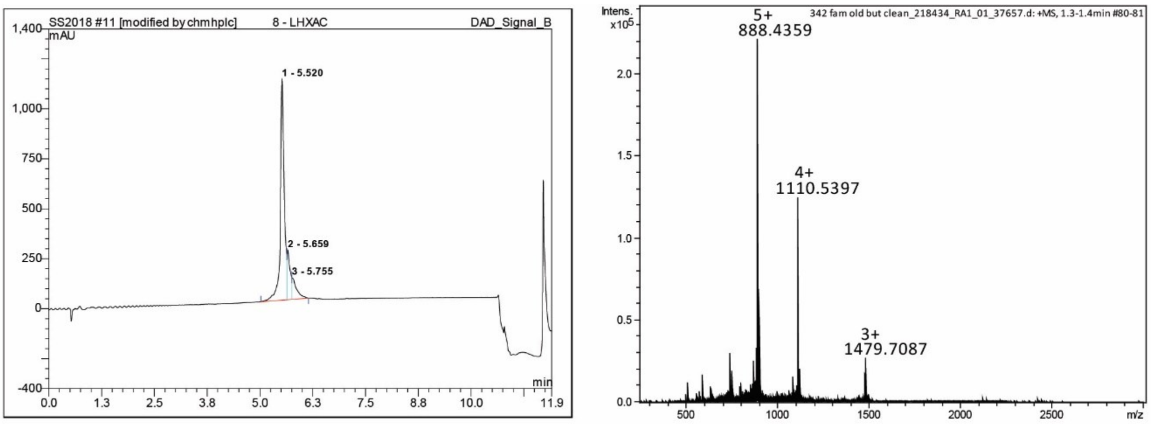

*h*DMX$_{333\text{-}373}^{pSer342/pSer367}$

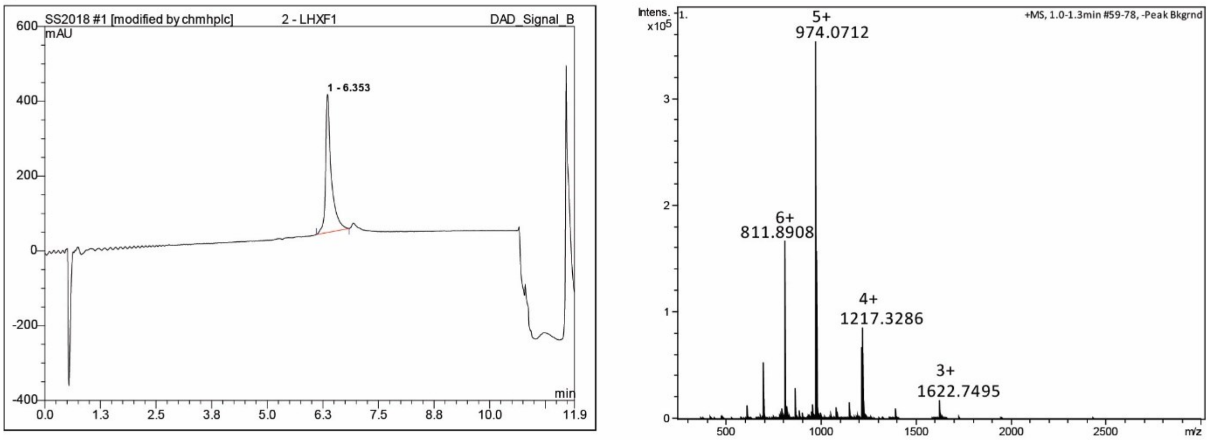

FAM-Ahx *h*DMX$_{333\text{-}373}^{pSer342/pSer367}$

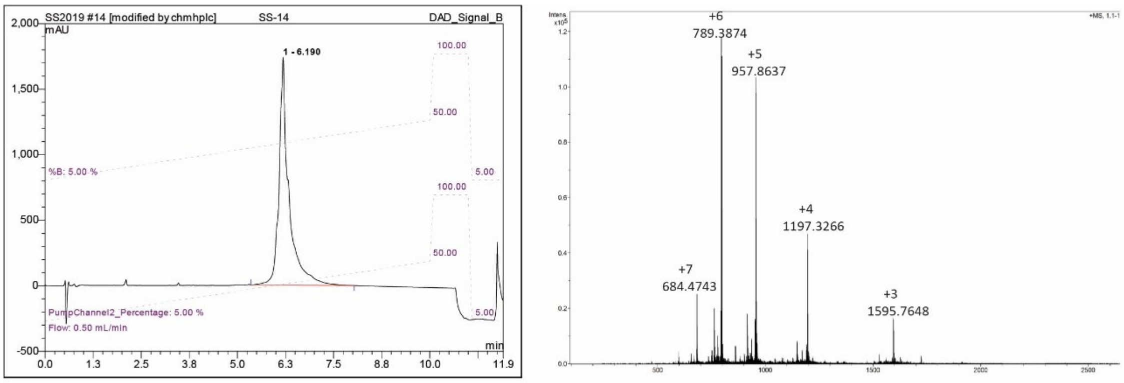

FAM-Ahx hDMX$_{333\text{-}373}^{pSer342}$

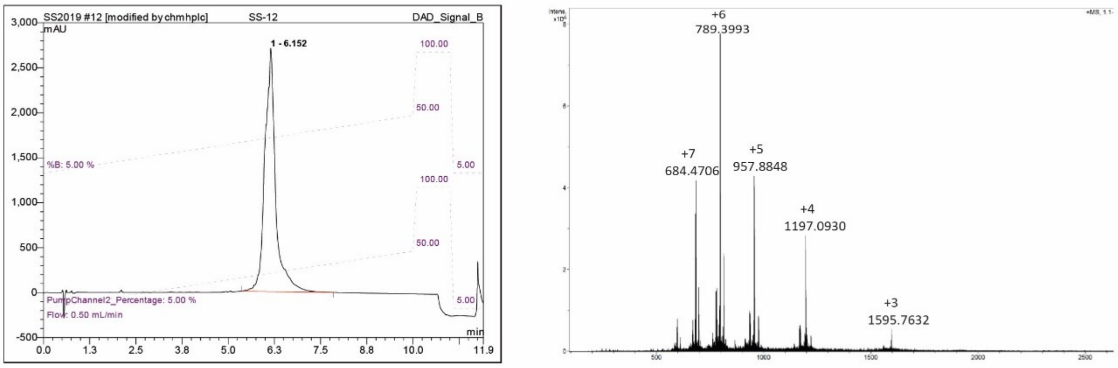

FAM-Ahx hDMX$_{333\text{-}373}^{pSer367}$

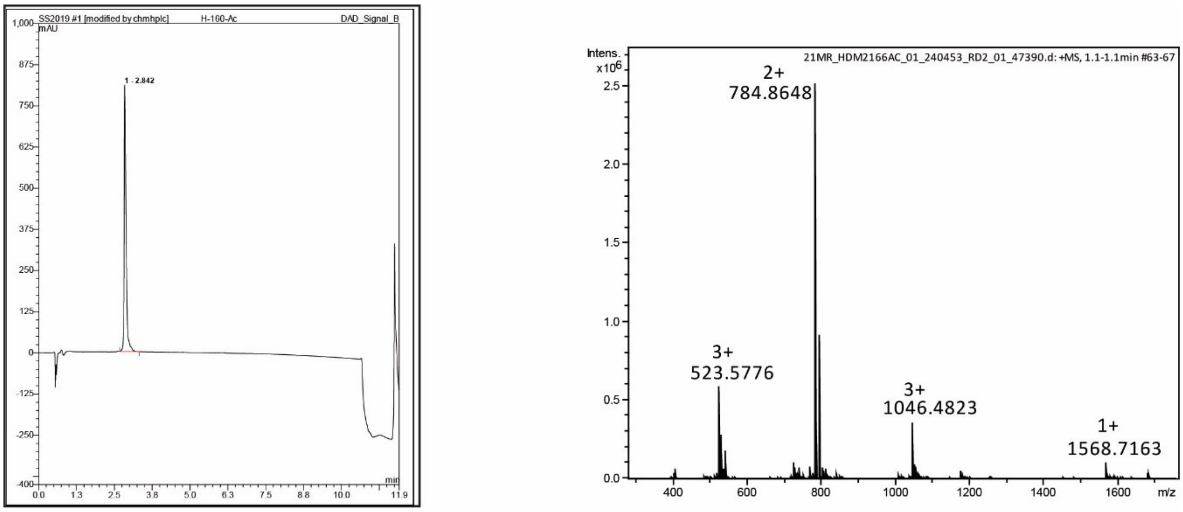

*h*DM2$_{160\text{-}171}$^{pSer166}

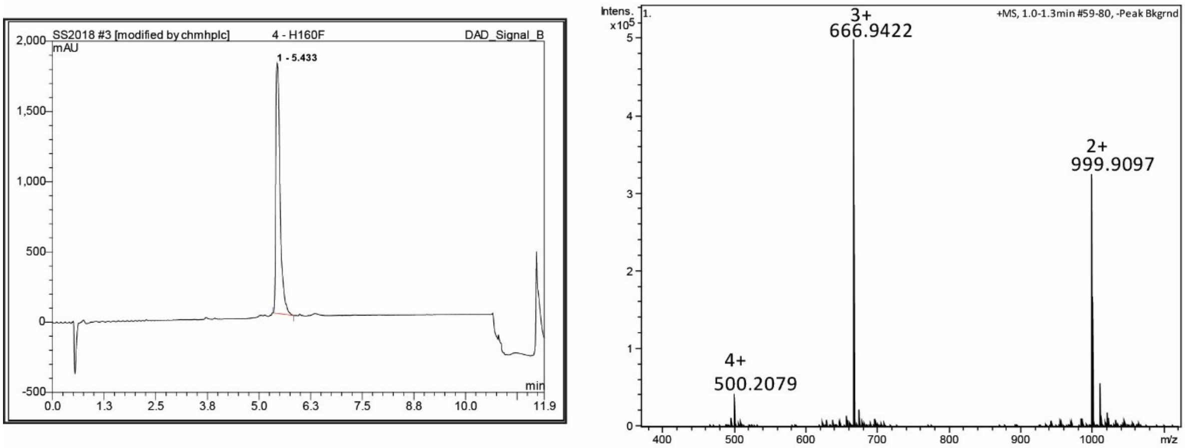

FAM-Ahx *h*DM2$_{160\text{-}171}$^{pSer166}

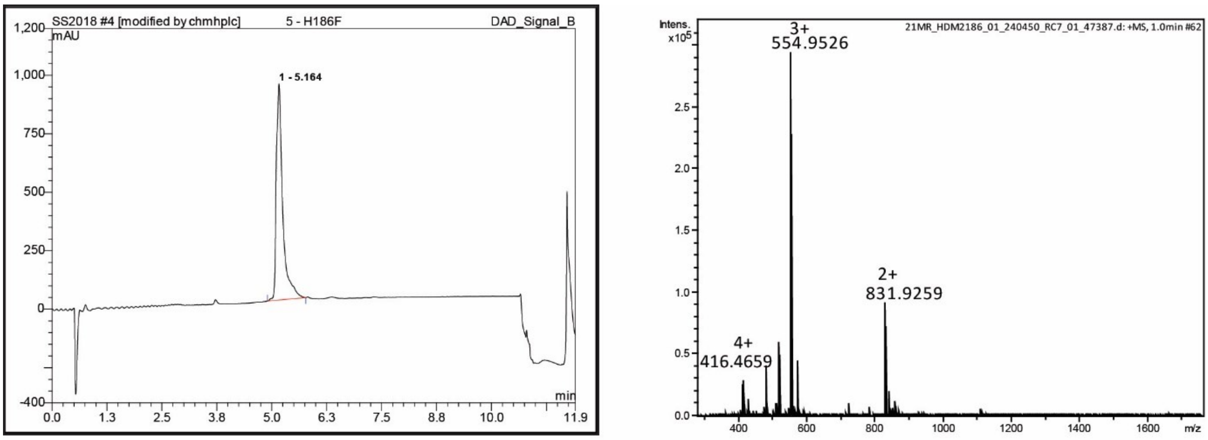

*h*DM2$_{180-192}^{pSer186}$

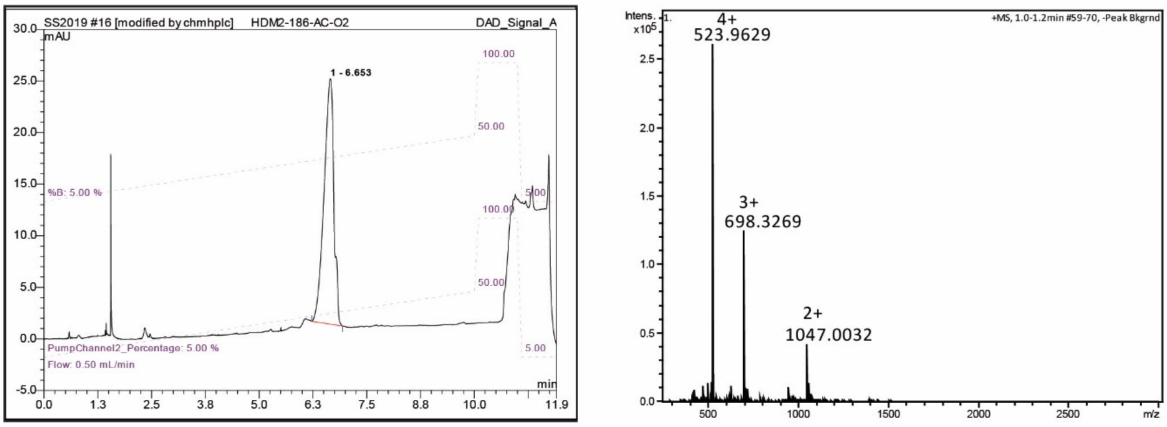

FAM-Ahx *h*DM2$_{180-192}^{pSer186}$

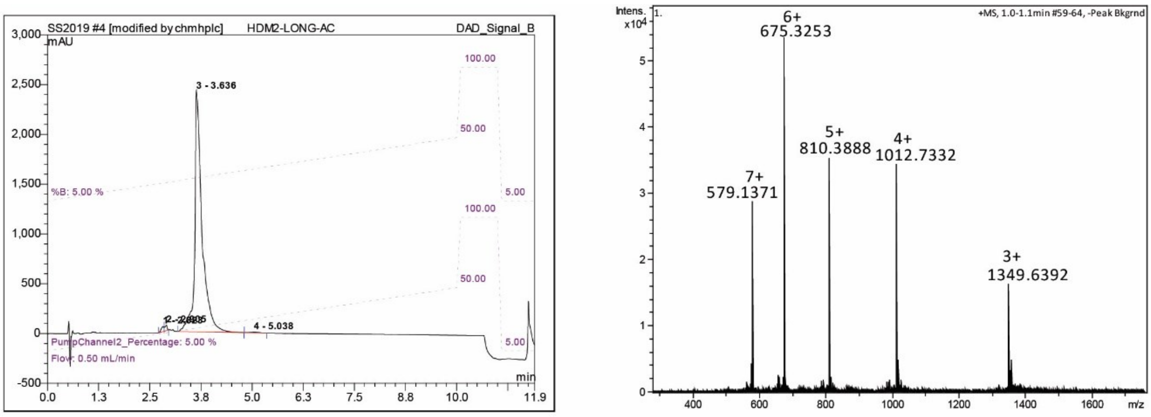

hDM2$_{160\text{-}192}^{pSer166/pSer186}$

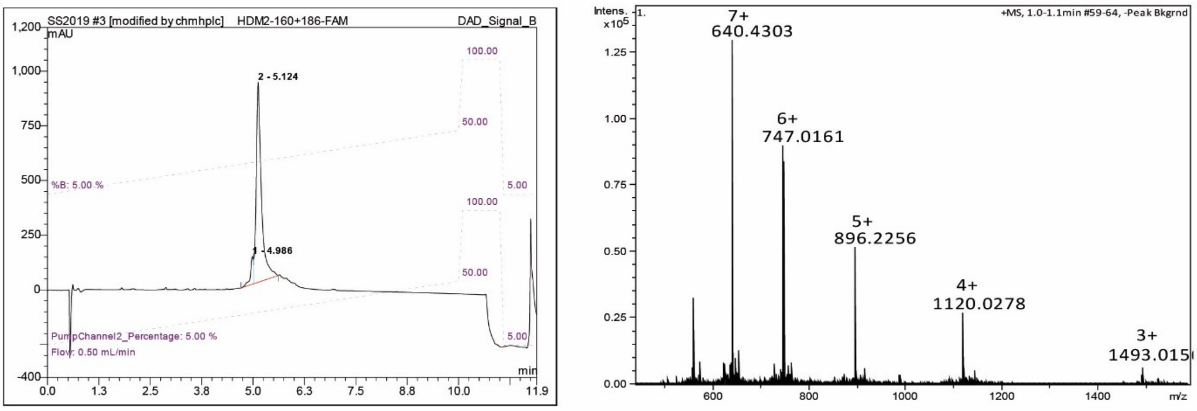

FAM-Ahx hDM2$_{160\text{-}192}^{pSer166/pSer186}$

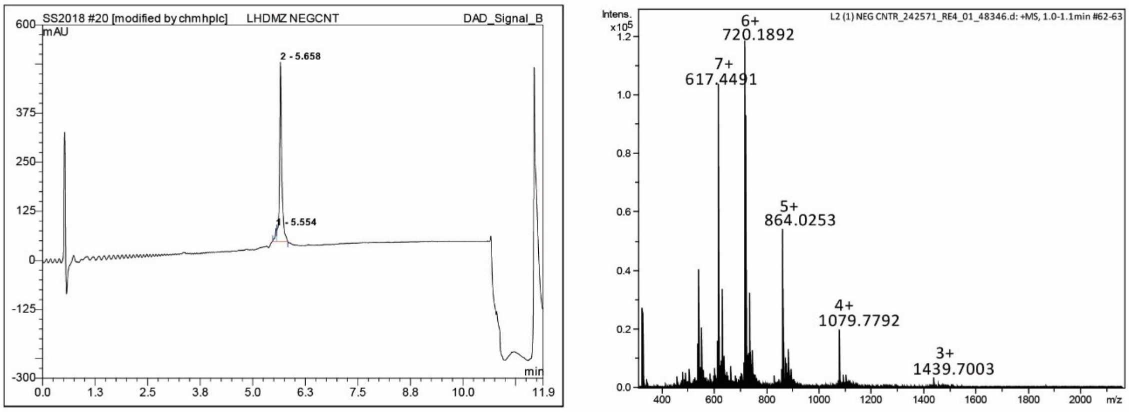

FAM-Ahx hDM2$_{160\text{-}192}^{pSer166/pSer186}$

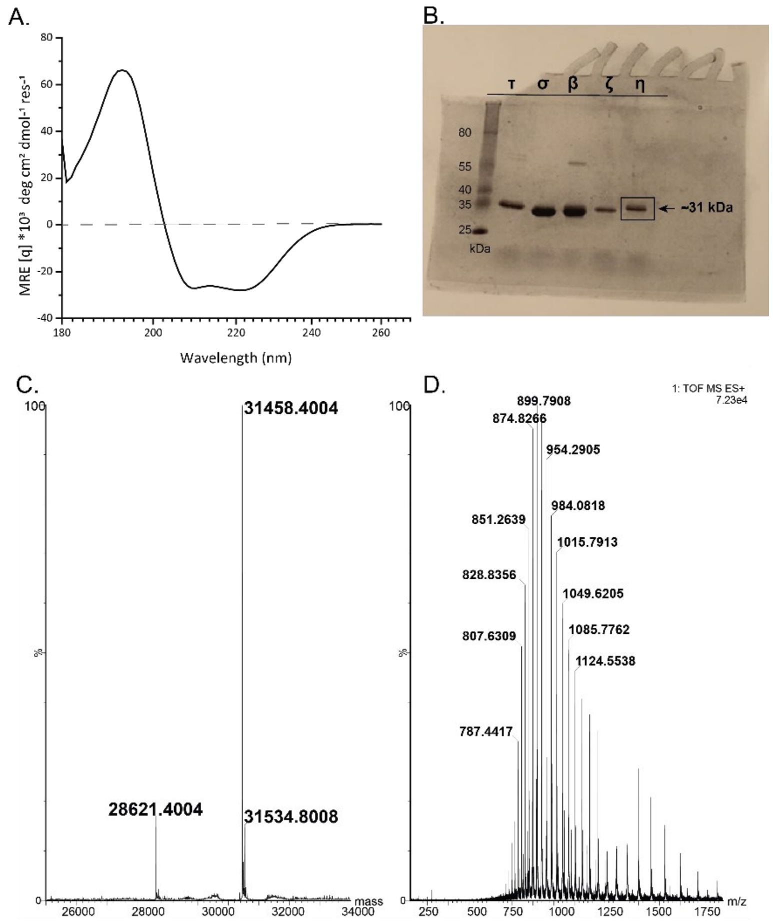

Representative 14-3-3 characterization and quality control (shown for 14-3-3η) (A) CD spectra showing an α-helical structure of the 14-3-3η (71%, theoretical helicity 78%, at 20 ℃, 50 mM sodium phosphate buffer, pH 7.5) (B) Coomassie Brilliant Blue stained SDS-PAGE gel after the protein purification C) Deconvoluted spectrum of 14-3-3η (D) QTOF MS showing a mass of 31 458.40 kDa (expected: 31 459, mass of 28 621.40 kDa corresponds to 14-3-3η without His_6-tag and the mass of 31 534.80 Da corresponds to addition of mercaptoethanol

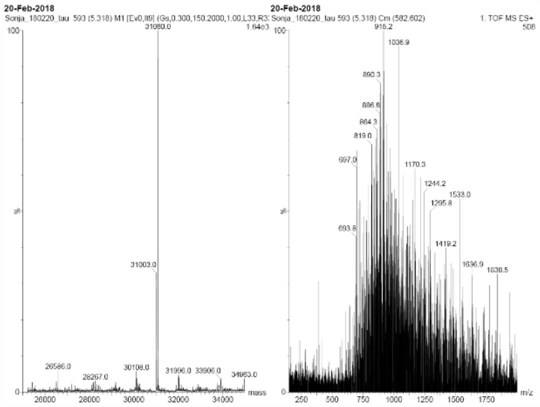

14-3-3 TAU
Mw=31005

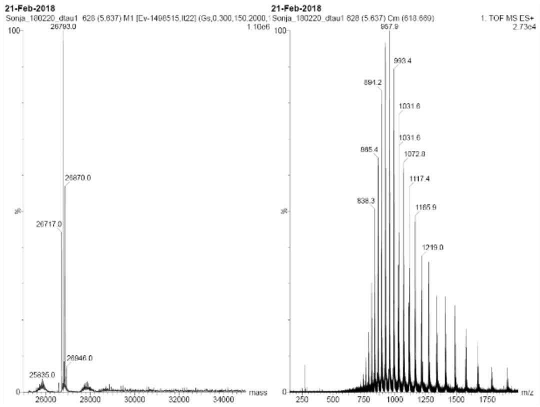

14-3-3 TAU DELTA C
Mw= 26718.3

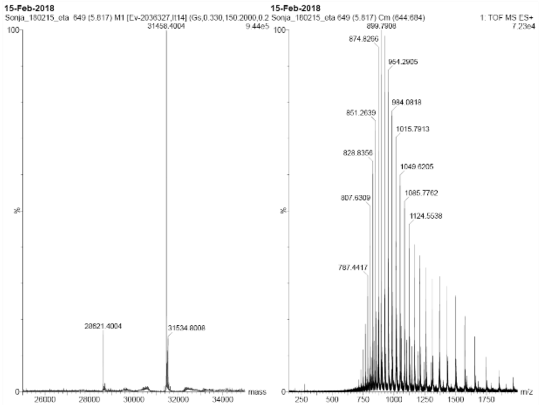

14-3-3 ETA
Mw= 31459

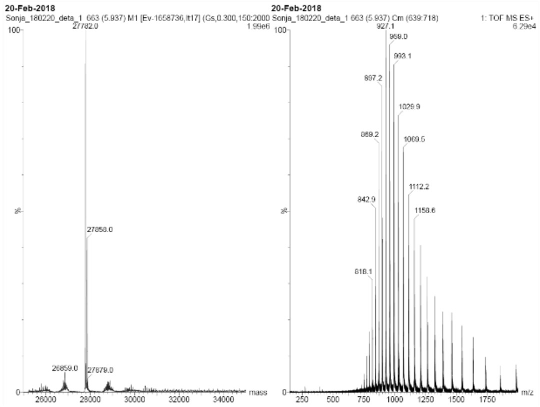

14-3-3 ETA DELTA C
Mw= 27782.4

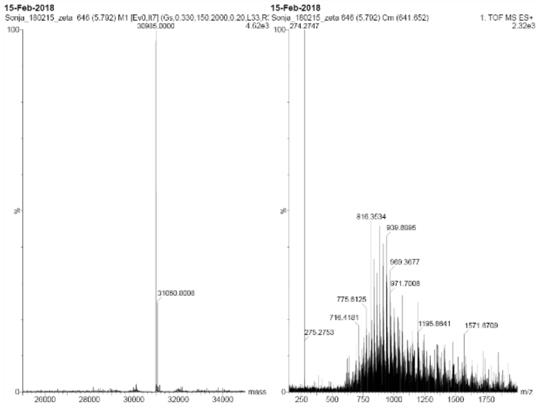

14-3-3 ZETA
Mw= 30985,5

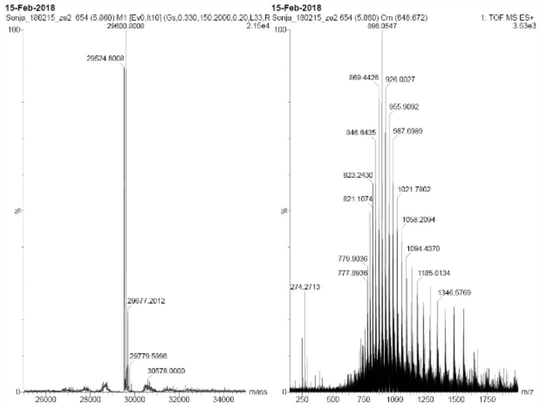

14-3-3 ZETA DELTA C
Mw= 26688.2

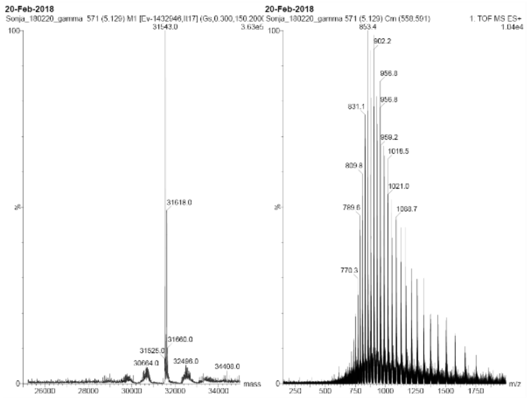

14-3-3 GAMMA
Mw= 31543

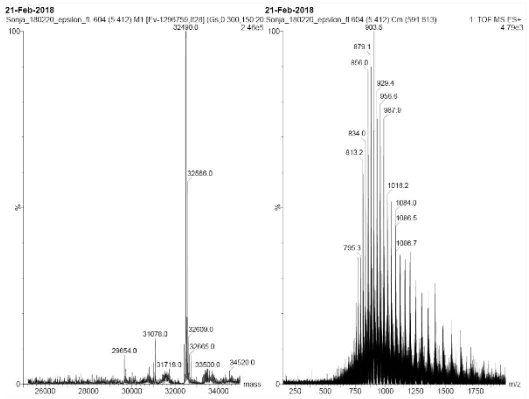

14-3-3 EPSILON
Mw= 32414

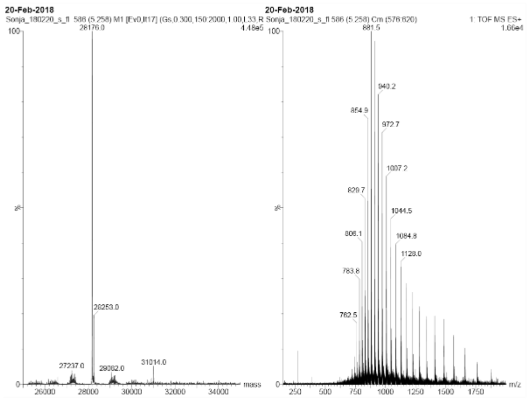

14-3-3 SIGMA
Mw= 31014.4
Major peak is 14-3-3 without his-tag

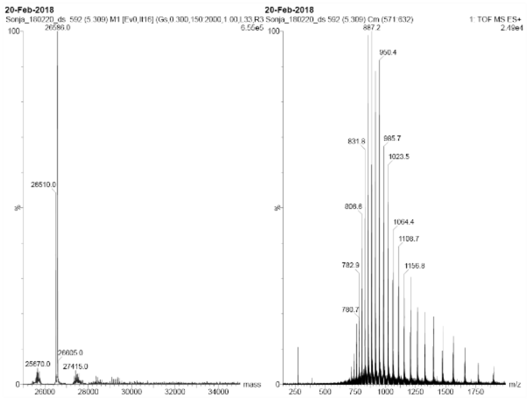

14-3-3 SIGMA DELTA C
Mw=26509.9

References

1. Madeira, F., Park, Y. m., Lee, J., Buso, N., Gur, T., Madhusoodanan, N., Basutkar, P., Tivey, A. R. N., Potter, S. C., Finn, R. D. & Lopez, R. (2019) The EMBL-EBI search and sequence analysis tools APIs in 2019, *Nucleic Acids Res.* **47**, W636-W641.
2. Scheuermann, T. H. & Brautigam, C. A. (2015) High-precision, automated integration of multiple isothermal titration calorimetric thermograms: New features of NITPIC, *Methods.* **76**, 87-98.
3. Schrodinger, LLC (2015) The PyMOL Molecular Graphics System, Version 1.8 in
